# Periorbital swelling in emergency room: Get your eyes in

**DOI:** 10.4103/0974-2700.55349

**Published:** 2009

**Authors:** S SenthilKumaran, N Balamurgan, K Arthanari, P Thirumalaikolundusubramanian

**Affiliations:** Department of Accident, Emergency and Critical Care Medicine, Sri Gokulam Hospital and Research Institute, Salem, India

**Keywords:** Facial swelling, headache, eye pain

## Abstract

Facial swelling and eye pain are very common patient complaints in Emergency Departments. Clinical evidence and investigations play a crucial role in making the correct diagnosis which impacts the final disposition and management of the patient. We present a case of a patient who presented with facial swelling and headache.

A 48-year-old diabetic and normotensive male patient presented to the emergency department with severe headache and vomiting with sudden onset of right facial swelling. He gave history of head injury with epistaxis three weeks ago. On examination he was irritable, febrile and tachycardic. His right side ocular examination [Figure 1] revealed lid edema, narrow palpebral aperture, chemosis, and marked restriction of ocular motility out of proportion to the degree of proptosis. There was loss of pupillary light reflex. Fundus examination revealed low-grade optic disc edema with dilated retinal veins. His visual acuity was reduced. The contralateral eye was normal. In view of headache and eye swelling, a preliminary diagnosis of cavernous sinus thrombosis was made and later the patient was subjected to MRI to confirm the same.

**Figure 1:**
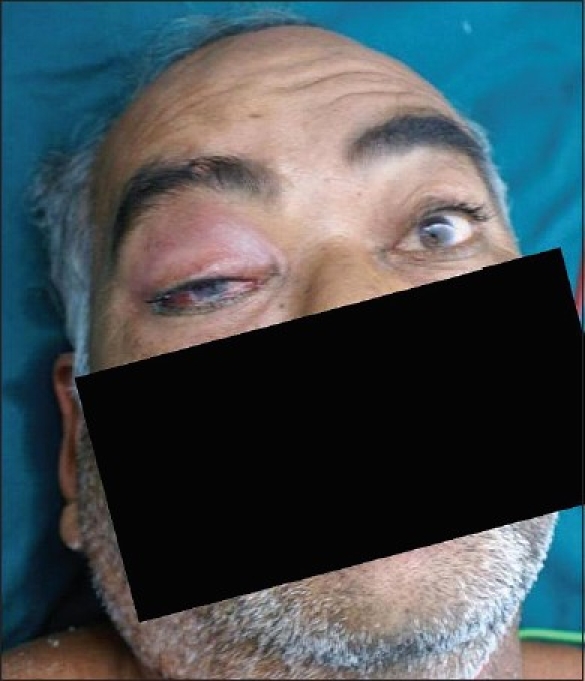
Clinical photograph

Contrast-enhanced MRI [Figure 2] demonstrated a meningeal enhancement consistent with meningitis, swollen non-enhancing cavernous sinus and narrowed internal carotid artery; this was consistent with the diagnosis of cavernous sinus thrombosis (CVT).

**Figure 2:**
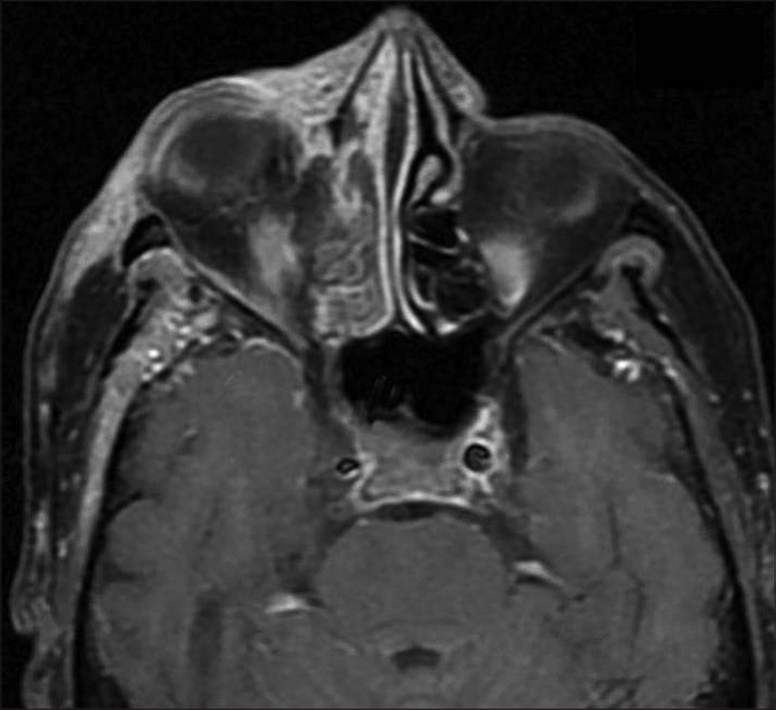
MRI

## DISCUSSION

Cavernous sinus thrombosis commonly results from contiguous spread of infection from the sinuses or middle third of the face. Other causes include trauma to the face, dental abscess or orbital cellulites. Approximately 70% of infections are caused by Staphylococcus aureus.

Headache[1] is the most common presenting symptom and periorbital edema may be the earliest physical sign. Cavernous sinus thrombosis is a clinical diagnosis and laboratory studies are seldom specific; however, magnetic resonance imaging with magnetic resonance venogram[2] is the preferred imaging choice to confirm its presence and to differentiate it from alternatives, such as orbital cellulites, acute angle closure glaucoma and carotid artery fistula. The mainstay of therapy is early and aggressive intravenous antimicrobial administration. Corticosteroids may help to reduce inflammation and edema. The use of anticoagulation for CVT is still a controversy.[3] Cavernous sinus thrombosis should be treated as a relative emergency as they have fulminant course with high rates of morbidity and mortality.

## CONCLUSION

The case summarizes success secondary to a high index of suspicion based on the clinical examination and results from imaging. CVT management if done in time can heavily impact positive outcomes.
